# Characterizing ‘Atmosphere’: exploring determinants of regular service attendance amongst integrated supervised consumption site clients in Vancouver’s Downtown Eastside

**DOI:** 10.1186/s12954-025-01272-2

**Published:** 2025-11-29

**Authors:** Benjamin D. Scher, Benjamin W. Chrisinger, David K. Humphreys, Gillian W. Shorter

**Affiliations:** 1 Department of Social Policy and Intervention, University of Oxford, Oxford, UK; 2 Department of Community Health, Tufts University, Medford, Massachusetts, UK; 3 School of Psychology, Queen’s University Belfast, Belfast, UK; 4 TreAdd Research Group on Treatment and Addictions, Tampere University, Tampere, Finland; 5 School of Psychology, David Keir Building, Queen’s University Belfast, Belfast, BT9 1NN UK

## Abstract

**Background:**

Research has explored barriers and facilitators to supervised consumption sites (SCS) in Canadian settings. Despite this, little is known about what factors drive individuals to initiate and repeatedly attend specific SCS where multiple SCS options are available, such as in Vancouver’s Downtown Eastside (DTES). The aim of this study was to understand the structural, contextual, and operational determinants of regular SCS attendance during Canada's ongoing overdose crisis.

**Methods:**

Rapid-ethnographic fieldwork was conducted over a six-week period at an integrated SCS in Vancouver’s DTES. This comprised an initial five-week period of non-participant observation (≈200 h) followed by a community consultation regarding the research design and question protocols. Qualitative data were then collected through five focus groups (n = 25) and 20 semi-structured interviews with regular service attendees with data analysed using thematic analysis.

**Results:**

Our findings highlight four important factors related to regular service attendance. The service had a regular clientele who described their attendance as routinized, which they attributed to four distinct factors: (1) the accessible location, (2) the on-site auxiliary health and support services, (3) the diversity of harm reduction provision, and (4) the atmosphere. Exploring the concept of atmosphere in more depth showed that it was characterized by the safety, familiarity, and inclusivity experienced within the service. Together, these factors facilitated a strong sense of belonging among attendees regarding the service and its community of staff and clients.

**Conclusion:**

Within the context of an ongoing overdose crisis, our findings highlight SCS service characteristics which facilitate routinized engagement including accessibility, wrap-around support, comprehensive and tailored harm reduction, and an inclusive welcoming atmosphere. These insights can inform policy makers and service providers in scaling and developing effective, client-centred SCSs to reduce harm and promote wellbeing.

## Introduction

In 2023, there were a total of 7162 drug-related deaths in Canada [1], placing it third in the world for overdose deaths per million, just behind the United States and Scotland [2]. The province of British Columbia (BC) has experienced approximately a third of the country’s deaths despite it representing only 13% of the national population [3]. Within BC, Vancouver stands out. With overdose rates surpassing those of any other Canadian city, Vancouver’s Downtown Eastside (DTES) has been coined by many as the epicentre of Canada’s opioid crisis and Canada’s poorest area code [4].

The DTES is a unique location in the context of the Canadian overdose crisis. As Vancouver’s oldest neighbourhood, this area was historically home to the cities original industrial economy, nightlife, hotels, and government buildings. Despite this early success, the DTES experienced economic decline as industry migrated to more affordable areas away from the downtown core [5]. The cost of housing in Vancouver is high, the DTES comprises the majority of Vancouver’s affordable housing, homeless shelters, and social and drug treatment services. As such, the DTES is home to Vancouver’s most marginalised and structurally vulnerable citizens [6]. It is more ethnically diverse (including an overrepresentation of indigenous residents), male dominated, older, and with fewer financial resources than anywhere else in Vancouver and is home to most of Vancouver’s homeless population [7]. The DTES has experienced some of Canada’s most severe forms of neighbourhood policing [4, 8, 9] and gentrification [10, 11] as municipal and provincial governments have sought to control and shape the visibility of poverty in its public spaces often without providing the resources to alleviate it. Local groups such as the Vancouver Area Network of People Who Use Drugs and the Pivot Legal Society have led successful advocacy campaigns for local and national reforms. For example, notable amongst those in 2003 with the legal sanctioning of Canada’s first supervised consumption site (SCS), the Vancouver based INSITE facility [12].

Evaluations of SCSs often focus on their effectiveness as interventions to reduce adverse health and public order outcomes [13, 14]. These studies provide a key role in summarising effects and addressing public and political opposition, including efforts to harmonise data across jurisdictions [15, 16]. However, it is also important to understand how these sites are experienced in practice [14, 17, 18]. Everyday interactions, relationships, and the social and material organization of these spaces remain relatively underexplored in the literature and undervalued in the policy realm.

Whilst many SCSs are the only harm reduction intervention of their kind in a given area, little is known about how individuals make choices when multiple SCS options are available. This may expand our understanding of how and why SCSs are effective in positively impacting clients'well-being [19–21] with regular use often linked to positive outcomes [14]. There have been theoretical explorations of the material and relational dynamics and organization of other settings (e.g., hospital setting interventions or retail environments) [22] and/or explorations of the role of staff e.g. [23]. In these studies, the recognition of the importance of *atmosphere* (the pervading character, feeling, or mood of a place or situation) on service quality has shaped the real-life environmental design of these interventions. An increased focus on these safe and supportive aspects of SCSs could shape our understanding of how SCSs improve service delivery [24].

Despite being a relatively small neighbourhood of approximately 4 km2, there are nine SCSs in the DTES [25]. These services operate through a range of models, from more medicalized sites such as INSITE, which contains clinical staff and on-site primary care, drug treatment and social services, through to lower-barrier services such as the SisterSquare Tent which is a peer-run supervised injection and smoke tent. Services also vary in their permitted modes of drug consumption; some allow for either injecting or smoking or a combination of the two. Whilst extensive research has evaluated the general barriers and facilitators to attendance, little is known about what factors drive individuals to initiate engagement and continue engaging with SCS services in socioenvironmental contexts, like the DTES, where multiple SCS options are available.

Here, we explore how SCS clients conceptualize their relationship to a specific SCS and the wider facility in which it is embedded. Although previous research has acknowledged that many SCS have a core group of regular clientele [2, 4, 26], the dynamics of these relationships have not been explicitly studied. By focusing on client-service dynamics this research seeks to advance our understanding of client decision making and the specific aspects of SCSs which foster sustained engagement and support inclusion health outcomes [15, 27, 28].

## Methods

### Study setting

This study took place at a low-barrier, harm reduction day centre in the DTES, which we anonymize to the Resource Centre (RC). It hosts a six-booth supervised injection room staffed by two health workers stationed at a desk at the back of the room adjacent to the booths. The room is decorated with artwork made by clients and posters with harm reduction advice. There are two doors; an entrance door which opens onto the front of the facility and an exit door which connects the SCS to the common room. In the back there is a supervised inhalation ‘tent’, staffed by a peer worker, which can accommodate six people. Although called a tent, it is a gazebo, with two of the four sides open to allow for ventilation, a table, six chairs and naloxone, an oxygen machine, and a juice and water station.

The facility also has a large common room with eight large circular tables where people relax and spend time once they have visited one of the consumption spaces. Here, people can watch TV, play board games, card games and sit during the four meals cooked fresh in the on-site kitchen and served throughout the day. There is a reception desk where clients can seek advice, access services on offer, and access harm reduction equipment. There are on-site washing machines, showers, and toilets. Once a week there is also a pro-bono legal advice clinic. The staff team is made up of both peers and harm reduction staff with approximately equal split. The facility encourages clients to become involved with the peer worker program as well as the twice-daily job draw, where any client can put themselves forward to complete small jobs around the facility in exchange for cash that same day. Operating hours of the RC are 9 a.m.–9 p.m. Observations and fieldwork occurred throughout the entirety of the facility.

### Overview of methods

To explore how SCS clients conceptualize their relationship to the SCS and broader RC community, data collection comprised six weeks of rapid ethnographic fieldwork at the RC. Rapid ethnography is a research approach that takes place over a shorter period than traditional ethnographies (usually around 90 days or less) and focuses on capturing social, cultural, and behavioural insights through multiple qualitative methods and the prioritization of researcher reflexivity [29]. It involves engaging a range of implicated stakeholders and triangulation in the analysis. This included: (1) a community consultation (n = 1), (2) five weeks (≈200 h) of participant observation and fieldnotes, (3) focus groups with regular clients (n = 5), (4) informal rapid-ethnographic interviews with clients (n = 20), and (5) semi-structured interviews with staff, including management and peer workers (although staff perspectives will be reported elsewhere) (n = 15).

### Research team and partnerships

Fieldwork and data collection was conducted by BDS who had previously worked for the host organization between 2019 and 2021 both in the RC and in other of the organizations housing and harm reduction services. DKH, BWC, and GWS supervised the project, contributing to design, analysis, and write-up, and although not present in Vancouver, met regularly with BDS online to discuss fieldwork and emerging findings.

### Recruitment, sampling and compensation

Recruitment for focus groups was done through a combination of snowball and purposeful sampling [30]. During the initial five weeks of participant observation BDS immersed himself within the day-to-day operations of the RC, in the consumption spaces and common room where participants would go to relax and socialize once they had consumed drugs. During this period, he built rapport with clients and discussed the scope of the project and upcoming focus group dates with eligible participants. People who signed up were also encouraged to discuss the study with eligible members of their peer network. Eligibility was defined as: (1) aged 18 + years (2) used the RC on a regular basis, (3) provided informed consent and (4) could speak English. Interview eligibility matched focus groups. Participants received $15 reimbursement to participate in the community consultation and focus groups and $10 to participate in an ethnographic interview. Prior to all focus groups and interviews, the scope of the study and details of participation was explained, participants had the opportunity to ask questions, they were informed that they could end their participation at any time and written consent was sought for all participants.

### Participant observation, community consultation and data collection

Fieldwork began with four weeks of participant observation conducted by BDS, who Monday to Friday, 9am-5 pm, immersed himself within the day-today operations of the facility. Across the common areas, the smoke tent and waiting area of the injecting room, he engaged in informal discussions with clients as a means of gaining contextual insights as well as building rapport with clients prior to the start of the formal methods of data collection. Fieldnotes were hand recorded from memory directly following conversations. This was purposeful as a means of not disrupting the natural flow of conversations [31]. These initial fieldnotes did not record direct quotes but recorded general contents of conversations and reflections from the researcher with regards to the social dynamics of the facility. This initial participant observation phase helped ground the subsequent more formal methods of data collection. In terms of positionality, BDS is a white doctoral student in his late twenties without lived experience of homelessness or harm reduction service access. Although entering this period of research as an outsider, he was an employee of the host organisation as a harm reduction worker and had previously worked in the RC for a two year period, three years prior to the research commencing. He had also previously conducted ethnographic research within this facility and he had existing rapport with many of the regular clients, staff, and peers of the service. He has received robust training in qualitative and ethnographic methods.

During the fifth week of participant observation, BDS facilitated a community consultation with six clients. Here, the group discussed the research design and question protocols, and feedback was given to ensure the questions were correct given the scope and aims of the study and that these questions were being asked in a trauma-informed manner [32]. Focus groups and interviews were conducted over the final two-week period. Multiple methods of qualitative data collection afforded triangulation of findings [33].

Five focus groups were conducted which lasted 40–60 min and contained four to six people per group (total n = 25). Participants were aged 30–56 years (Mean = 52; SD = 10.3). Seven participants self-identified as Indigenous and 18 self-identified as White. There were two females, 22 males and one who self-identified as non-binary. Seven were housed, eight in temporary or emergency shelter accommodation, and 10 self-identified as unhoused. Focus groups took place in a private room in the building next door to the RC which was a housing facility operated by the host organization. The question protocol was semi-structured, and participants were asked questions related to their experiences using the supervision consumption and other services at the RC, their views on operational policies, their experiences with other local harm reduction services and the impact they believed the RC had in their lives. These included questions such as: “How often and why do you come to the RC?”, “How would you describe your relationship with staff?”, “Which services within the facility do you tend to use?”, “How would you compare the RC to other similar services in the DTES?” and “Has the RC impacted your life beyond the immediate harm reduction benefits of consuming drugs whilst supervised?”. Participants were recruited by BDS from the SCS room and the smoke tent. Following initial engagement, BDS would accompany participants next door to the private meeting room. Once he had the desired number of people, he explained the scope of the project, assisted people in completing the consent forms and demographic questionnaires. Focus groups were audio recorded for subsequent transcription. Snacks, and soft drinks were provided during the sessions.

One-on-one interviews generate different responses to focus groups–this is especially true in qualitative research with vulnerable groups who may not feel comfortable opening up in group settings or who may be influenced by peer responses [26]. Using both methods to triangulate findings offers a more complete picture of the topic of interest [34]. Rapid-ethnographic interviews were conducted by BDS, who took handwritten fieldnotes and recorded verbatim quotes of importance. These quotes were then read back to participants at the end of interviews to make sure they accurately reflected the meaning of what they were conveying. Individual interviews were semi-structured and followed a question protocol relating to their experiences in the RC, their perceptions of other local services and their relationship with peers and staff. Questions included: “How does your attendance to the RC fit into your daily routine?”, “What are your main reasons for coming to the RC?”, and “Is there every anything that happens here which you are unhappy with or that you would like changed?”. These interviews lasted between 5 and 15 min and took place in the RC in an area of the common room or courtyard away from other clients. Due to these interview sessions being done relatively quickly and to ensure anonymity, we only gathered limited demographic data related to gender and ethnicity. The sample included three women and 17 men with five self-identifying as Indigenous, one as Black, one as Asian and 13 as White (total n = 20).

### Ethics

Ethical approval for this study was granted by the University of Oxford Central Ethics Committee on 11/03/2023 (Ref: R84228/RE004) and internal ethical approval from the host organisation. The approved, anonymised ethics protocol is hosted on the Open Science Framework (DOI: 10.17605/osf.io/vs4at). To ensure the confidentiality and anonymity of participants, individual names were omitted from this article and were not associated with individual quotations [35]. During focus groups and ethnographic interviews, staff were not present, and could not hear what was being said.

### Data analysis

All audio and handwritten data was transcribed and uploaded to NVivo 15 qualitative analysis software which allowed researchers to collaborate on the coding process. Subsequently, data analysis comprised of the Braun and Clarke [36] six stage reflexive thematic analysis which commenced with a process of familiarization with the data, whereby BDS read transcripts and fieldnotes, wrote analytic memos, and developed a thematic codebook to work systematically through the data, identifying relevant and meaningful information related to our research questions, as well as novel concepts inductively. Following this initial coding, BDS and GWS re-examined transcripts in the context of this initial coding, iteratively refining the code book. All authors refined the final coding framework [37].

## Results

We present our results under three central themes (see Table 1), each with sub-themes related to the narratives, experiences, and perspectives of clients in relation to the RC, its impact in their lives, and the dominant determinants of service utilization:

**Table 1:** Themes and sub-themes of the views of RC clients

Theme	Sub-Theme
1. Routine and Regular Attendance	
2. Determinants of Service Utilization	2.1 Location
	2.2 Diversity of harm reduction provision
	2.3 Integration of services
3. Characterizing ‘atmosphere’	3.1 Safety
	3.2 Familiarity
	3.3 Inclusivity
	3.4 Belonging

**(1) Routine and regular attendance**

From the start of fieldwork, two aspects of the SCS were visibly apparent. First, the service was in high demand and as a result both the consumption spaces (injection room and smoke tent) and common area, were typically busy from the very start of the day until closure. Secondly, it appeared most clients attended the service daily. Fieldnotes frequently highlighted the busyness of the service, an observation corroborated by participants:

*“You’d be amazed at how this place helps. It’s always packed and it’s an amazing place for the population to get help, both day and night.” (Interview Participant 6, White, Male)*

The high demand speaks to the impact the service has in the lives of its clients. For many, attending the SCS was less about sporadic visits centred purely around harm reduction, but as a space which helped establish a routine and daily structure, as this participant explained:

*“For me I go there in the morning and get my coffee and then at one o'clock, you got a bit of food they give out and I appreciate that too…it's at a set time and it's always there.” (Focus Group Participant 18, White, Male, 58 years old, Unhoused)*

Many participants highlighted the predictability of the timings of the service. This was echoed by participants who described the service as a hub for other structured, meaningful activities that helped them establish routine and take part in activities they enjoyed:

*“I like it just because nine in the morning until nine in the afternoon, I go in there and I can grab a rag and spray bottle and can wash my bike and just do things to keep busy.” (Focus Group Participant 24, White, Male, 41 years old, Temporary Accommodation/Shelter)*

Descriptions related to the value placed on routinized attendance was common: *“I think it is routine, especially for coffee and football on Sundays.” (Focus Group Participant 23, White, Male, 52 years old, Housed)*. For many, being able to come to the RC acted as an anchor in their daily lives. As was recorded in depth in the fieldnotes, many participants chose to solely access this facility despite the wide range of comparable services on offer in the DTES. This was exemplified through comments such as *“I am homeless in a tent but me and my buddies are sensible with it… I use here exclusively.” (Interview Participant 6, White, Male).* Highlighted in this theme and reoccurring throughout data collection was the routinized, daily and exclusive attendance of this service versus the others available locally.

**(2) Determinants of service utilization**

### Location

Participants consistently spoke about the importance of the services’ location, repeatedly highlighting this as a key reason why they chose to access this particular SCS. Often, proximity to where people lived, worked, and accessed other services were important practical consideration:

*“I live and work next door, so this is really convenient.” (Interview Participant 7, Indigenous, Male)*

Convenience in relation to peoples’ residence and other routinized daily activities like work or health care access was repeatedly highlighted:

*“It’s a good location, it’s close to my pharmacy and where I live. I come here every day really.” (Interview Participant 16, Black, Male)*

The usefulness of the proximity of the service to other convenient, used services and structures was also narrated by people who have mobility issues. For example, one service user stated:

*“It’s very close to where I stay and I really struggle to get around.” (Interview Participant 2, Indigenous, Male)*

In addition to reflections on spatial and temporal needs, the location of the service was discussed in relation to its relative distance from the central core of the DTES. For some it was far away from structures and settings they wanted to avoid; that the service was slightly outside of this core was seen as a positive although they did not give a reason as to why:

*“It allows you to get away from it all. It's a little bit outside that main core, right?” (Focus Group Participant 13, White, Female, 37 years old, Temporary Accommodation/Shelter)*

This sentiment was highlighted by an additional participant who was explicit about the perceived benefits of being outside of the neighbourhood core. In this case, it was about being separate from others, about safety, and about ‘stuff’ they did not want to be exposed to:

*“It's off the beaten path. It's not in the core of the DTES. This place is…a good couple of blocks away from the real hardcore stuff…it’s away from all that. So when you step out, you're not stepping over people you know.” (Focus Group Participant 19, Indigenous, Non-Binary, 54 years old, Temporary Accommodation/Shelter)*

This ‘stuff’ was elaborated to explain the higher concentration of people ‘nodding off’ (losing consciousness) or overdosing in street-based settings in the neighbourhood core and speaks to the impact of this daily reality.

### Diversity of harm reduction provision

Participants frequently highlighted the diversity of harm reduction services on offer, with many expressing how *“great the harm reduction services are here”* (Interview Participant 2, Indigenous, Male). The accommodation of people who smoked drugs was key to many people's regular attendance:

*“Without the smoke tent we would all be using alone. Everyone always focuses on injections but all my friends use the tent too.” (Interview Participant 9, Indigenous, Male)*

This sentiment was common, as seen in this focus group excerpt where the fact that this facility supports a wide range consumption practices is emphasized:

*Speaker 1 (Focus Group Participant 6, White, Male, 54 years old, Unhoused) – “This is actually a place where you can smoke…many of them don't, like INSITE [a more medicalized SCS in the DTES] doesn't….that makes a difference because lot of people like smoking…and to have a place…where you can do it…not worried. It's either that or the street…and the street is windy…for the smokers…it kind of sucks.”*

*Speaker 3 (Focus Group Participant 8, White, Male, 51 years old, Temporary Accommodation/Shelters) – “We have always had injection sites but for years we never had a place to smoke…smoking on the street is a lot but more of a hassle. They should have more around town.”*

*Speaker 9 (Focus Group Participant 9, Indigenous, Male, 55 years old, Unhoused) – “I've noticed since I've been down here almost 10 years. There's a lot more smokers than there are pokers…and so there's a greater need for smoking tents and a lot of people smoke their drug like me, that’s what I do exclusively.”*

The diversity of harm reduction provision at this facility was greatly valued. For these participants, the smoke tent met their practical harm reduction needs and symbolized a more inclusive model of harm reduction which provided for a wider group of people who use drugs, a place for ‘pokers’ and ‘smokers’.

### Integration of services

Participants highlighted the integrated nature of the SCS as a key motivator for regular attendance. The ability to access multiple resources in one location was particularly significant, especially for individuals experiencing homelessness or precarious housing:

*“I just found that it had the most resources that I could use in one place, and it's open the longest which surprised me when I came to Vancouver that there is no 24-hour shelter or drop-in centres. You get to a certain point, maybe 9 o’clock and then you're on your own…and you got to go hang outside in the cold, no matter what.” (Focus Group Participant 9, Indigenous, Male, 55 years old, Unhoused)*

Whilst this participant explains that other services do offer similar resources, opening times tended to be restrictive. For many, especially individuals experiencing homelessness, being able to get out of the cold and access basic necessities such as food, water and hygiene facilities at any time of the day was incredibly important and distinguished this service from others:

*“It’s warm, its inside, food, bathrooms, I live in a tent and don’t have those things and you get really grungy, it’s not nice really. My tent is clean but still this place really helps with that.” (Interview Participant 15, White, Female)*

These practical supports were not just critical to physical well-being but also to peoples’ maintained sense of dignity and mental well-being:

*“It’s great they give you a place to just sit and be. When I was homeless this place kept me alive. Small things, the laundry and the food make a huge difference. Gloves, hats, it’s the small things we don’t get from anywhere else.” (Interview Participant 4, Indigenous, Male)*

Finally, despite a high number of food banks and SCSs in the DTES, public laundry services are not common. This offering at the RC was described as significant: “*There's no laundry within 40 blocks…other than this building.” (Focus Group Participant 3, Indigenous, Male, 48 years old, Unhoused)*. This observation highlights a significant gap in the availability of basic amenities within the DTES and positions the RC as a unique and highly valued resource; however, other key issues include shelter from the weather, food provision, and the simplicity of having a place to be, and be accepted.

**(3) Characterizing ‘atmosphere’**

Participants frequently noted how the atmosphere of the service differed to that of other local services. Statements such as *“I like joining my friends and the atmosphere and people are generally nicer than down in the other places” (Interview Participant 19, White, Male),* were common. During focus groups and interviews, this was something that we probed further. Our analysis suggests that descriptions of atmosphere were characterized by the creation of a safe, familiar, and inclusive environment. This enabled participants to feel a strong sense of belonging to the facility which encouraged regular service engagement.

### Safety

When discussing the atmosphere of the service, participants frequently mentioned the importance of safety as a precondition for positive experiences in the facility:

*“This place is open to everyone, it’s safe, we can mingle and not feel stressed.” (Interview Participant 2, Indigenous, Male)*

Here, the participant framed safety as physical protection and an emotional state fostered by the space. The ability to"mingle"without stress suggests that the SCS facilitates social interactions in a way that contrasts with the chaotic (or threatening) environments participants may encounter elsewhere. However, as people were not concerned about it being open to everyone, this suggests it feels well managed as a space. The facility and the atmosphere in it were often framed in contrast to other environments they frequent in the DTES, acknowledging the many structural challenges faced by neighbourhood residents:

*“I stay this side of Main, there is too much chaos on the other side. There is more of a desperation to feed their addiction down there.” (Interview Participant 3, White, Male)*

In addition to interpersonal safety, participants conceptualized safety and the provision of harm reduction services in relation to their potential interactions with the police and the potential for criminalization:

*“It’s very thoughtful the SCS and the smoke tent. It does what's on the tin, you feel safe and aren’t being bugged by the cops*.” (Interview Participant 5, Indigenous, Male)

Despite the facility creating a place of safety and refuge, violence still occasionally occurred within the facility. This was described as severely disruptive to the atmosphere of the service and deterring attendance, at least temporarily:

*“Violence generally dissuades people so if there is a big fight it will be a bit more quiet the next day.” (Interview Participant 3, White, Male)*

In these situations, staff were able to instil trust in clients and re-establish order by swiftly and appropriately managing situations. This was seen as an important aspect of the safety of the facility and was again contrasted to how such situations are handled in other similar services:

*“I come here almost every day and it is the staff and the community which make it feel safe. Like yesterday there was a fight but staff got them out pretty quickly and the peers really help with that. At some of the others places, these incidents can drag out for hours.” (Interview Participant 20, Asian, Female)*

The value placed on the calm, swift, yet appropriate response from staff and peers was repeatedly emphasized by peers:

*“At the other places towards main (the neighbourhood core), people get bear sprayed and as a result the staff are just a lot more strict and on edge.” (Interview Participant 12, White, Female)*

These accounts underscore the role of staff and peers in diffusing conflict and restoring order. The efficient resolution of incidents was often contrasted with other services where conflicts either escalated or persisted. In addition to physical or emotional safety as a determinant for regular service access, safety also created a space where positive social experiences can occur:

*“It’s nice to be using around others, it’s just nice. You feel safe but it’s also more enjoyable.” (Interview Participant 6, White, Male)*

The emphasis on enjoyment suggests that the SCS fulfils pragmatic health needs of clients and emotional and social ones, providing a rare space that people who use drugs can occupy without fear of physical violence, stigma or apprehension from police.

### Familiarity

Familiarity with staff, other clients and the service emerged as a crucial factor in fostering a sense of comfort and trust among participants. The relationships developed overtime were frequently described as facilitating continued service engagement. Again, this aspect of the service was presented in contrast to other local services:

*“It’s the comfort, it’s the same people that come over and over so we feel safe, it’s comfort we don’t otherwise have.” (Interview Participant 18, White, Male)*

This sentiment was echoed by another participant who compared the community at the RC to that of a family:

*“It’s a couple of things but to me I agree it’s the atmosphere. It's basically a family, the same group people that come in every day. You see your face…you see the same people, it’s a core.” (Focus Group Participant 3, Indigenous, Male, 48 years old, Unhoused)*.

This quote highlights how repeated interactions with the same individuals can foster a sense of stability and predictability. For some, the facility served as a primary meeting place, enabling social connections, and alleviating social isolation common amongst people experiencing homelessness:

*“All my friends come here, it’s where we meet and hangout and eat because a lot of us aren’t able to provide it for ourselves.” (Interview Participant 4, Indigenous, Male)*

Being around familiar people also made people feel cared for in their immediate interactions. This contrasted to the experiences people had in street-based settings:

*“I know everybody and they know me and we watch out for each other as best as we can. We have a smoke but then watch TV and actually enjoy the high instead of just being kicked out on to the street.” (Interview Participant 18, White, Male)*

Although most clients used drugs and accessed one of, or both of the two supervised consumption spaces, there was a small portion of service attendees who accessed the common area exclusively. The peer support and familiarity extended to all clients:

*“I like all the different characters here and we all know each other. I mean I don’t use [drugs], I hate people doing it in front of me. I don’t like watching it but here we can all be together and not have to see it.” (Interview Participant 19, White, Male)*

In addition to peer relationships, participants consistently praised staff for their role in fostering a welcoming and approachable service. Familiarity with staff enhanced the sense of safety and comfort:

*“The staff here are more friendly and fair than [anonymized name of other local service being described], it feels less community [there]. Here there are more regulars, and you really get to know the other people that come here.” (Interview Participant 20, Asian, Female)*

The impact of staff in creating a welcoming environment, also extended to the role of peer workers, in particular their ability to foster a sense of trust and relatability with clients:

*“Having peers makes it much less intimidating. I know these guys and know that they are trustworthy.” (Interview Participant 7, Indigenous, Male)*

Familiarity in relation to fellow clients, staff and peer workers were all central in the creation of a welcoming environment where people felt comfortable, safe and trusting of the people around them.

### Inclusivity

Inclusivity, and more specifically the sense that the service was non-judgmental, everyone was welcome and that no one would be discriminated against was frequently highlighted as a key factor which encouraged attendance. One interviewee described the importance of not feeling pressured by external agendas, such as religious affiliations, which they had experienced at other services:

*“A lot of the other places are run by Catholics and this place doesn't push anything like that on us.” (Interview Participant 14, White, Male)*

This reflects how inclusivity is enhanced by neutrality in the service’s ethos, allowing individuals to access support without feeling alienated or judged. The non-punitive nature of the facility also emerged as significant, with participants expressing appreciation for its welcoming policies and the difficulty of being excluded:

*“Everyone here is friendly and it’s pretty hard to get kicked out.” (Interview Participant 4, Indigenous, Male)*

For some, the inclusivity extended to specific living circumstances, such as the challenges faced by couples experiencing homelessness:

*“It would affect me a lot if this place was shut down. It’s so hard to find couples shelters so we camp out back and use this place for all of our food, laundry and anything help wise really because at the moment the shelters won’t take us.” (Interview Participant 12, White, Female)*

This participant underscored the importance of inclusivity for marginalized groups, such as couples who are often excluded from shelter settings in the DTES or made to stay separately. Another recurring theme during interviews and focus groups was the minimal barriers to accessing basic resources within the RC:

*“I’m grateful because this is a place which is welcoming without questions. I can get food with no bullshit questions, a shower with no bullshit questions. I just prefer it that way.” (Interview Participant 14, White, Male)*

Inclusivity in the client group was evidenced through the diverse client group. Participants described how the service facilitates an environment which is welcoming for a wide array of marginalised groups, something other services struggled with:

*“There's different types of people that integrate really well here and that is only because they've worked hard to integrate well here. There are the hard, skilled drug users that understand the alcoholics and the non-drug users and the staff and it’s like each group…are all working all together. The staff are not overbearing, which happens at other places. The drug users are not too aggressive to the alcoholics that piss them off. The alcoholics are not too aggressive to the drug users. It's just everybody's integrated, softly, slowly and then it's worked well and everybody knows each other.” (Focus Group Participant 1, Indigenous, Male, 56 years old, Unhoused)*

These observations suggest that inclusivity is not just about providing a space but about cultivating an atmosphere and culture of mutual respect and understanding, supported by staff who can mediate tensions effectively. Finally, the service’s ability to accommodate pets, common amongst residents of the DTES, yet an uncommon policy feature in similar facilities, was important for participants whose pets were central to their well-being:

*“It is a dry place I can be with my dogs, most of them [other local SCS] don't allow dogs…my dogs are cold and I’m tired, and it’s a place to charge my phone, I'm sleeping in a car and I don't feel good and my car is full of mould and I need the place to recharge my brain I guess. I need hot water for tea. There's a lot of things…you need for a day. Honestly, if I had the chance to be with my family and my kids [I would be] but I can’t so this is where I can be right now and I choose this place because they love my dogs.” (Focus Group Participant 13, White, Female, 37 years old, Temporary Accommodation/Shelter)*

The service’s inclusivity stems from its ability to meet a wide range of needs without judgment or exclusion; and shows the difficulties people experience trying to meet their basic survival needs outside the centre. Intentional and proactive inclusivity worked to foster an environment where individuals felt respected, valued, and supported.

### Belonging

The combination of safety, familiarity and inclusivity created a space where individuals felt a genuine sense of belonging to both the service and the community within it. This characterization of the service as a “living room” underscores its role as a communal space where often marginalised individuals were able to relax:

*“It would affect a lot of people if this place closed. A lot of people would go astray. This is the living room for a lot of homeless people.” (Interview Participant 5, Indigenous, Male)*

Others described the facility as their second home:

*“This place is more open and welcoming, the staff, the people, I have nothing bad to say…It’s like my second home and for a lot of other people it is as well.” (Interview Participant 5, Indigenous, Male)*

These descriptions contrasted to how people referred to other services locally, with statements such as *“it feels more family oriented than the other's places, I feel more like a stranger in those spaces” (Focus Group Participant 11, White, Male, 64 years old, Housed)*. At the RC there was a real sense of collective solidarity amongst clients, which again contrasted the isolation often experienced in other settings they frequented:

*“People smoke out back, away from the street and we are all here together to save people. Here it is very much if today I have for me, I’ll give to him because I don’t know if I will have enough for tomorrow.” (Interview Participant 13, White, Male)*

Participants echoed these sentiments, describing in detail the sense of belonging they felt because of the long-standing relationships built within the facility:

*“It’s a safe place where people can be at to socialize, eat, wash, and just be. I have been coming here since 89’ and it’s the friends, my friends, the culture and the continuance of being a part of something and here it feels like that. We’ve all been through a lot here but this place appreciates us anyways.” (Interview Participant 17, White, Male)*

As a clear space for communal solidarity, the acceptance felt and sense of belonging described was often done in relation to the mutual hardships people had experienced. This final focus group excerpt encapsulated all of the points discussed above:

*Speaker 5 (Focus Group Participant 15, White, Male, 55 years old, Temporary Accommodation/Shelter) – “I consider it partly to be my home”.*

*Speaker 3 (Focus Group Participant 13, White, Female, 37 years old, Temporary Accommodation/Shelter) – “I guess it's the living room”.*

*Speaker 4 (Focus Group Participant 14, White, Male, 58 years old, Housed) – “Yes, like I said it’s a living room for a really dysfunctional family. And it's a safe place…even though shit happens…shit happens everywhere but here it’s a safe place…you get in fights with your dysfunctional family, but physically…it's pretty rare…mostly it's just verbal yelling and screaming and…in other places…you don't know the people because faces change and they don't go there daily…it seems like for a lot of people, it's a daily thing…they come get their morning coffee and they might leave and then they come back and or they might hang out for a couple of hours, watch the hockey game or watch the basketball game or the price is right…and then they take off and you know, for a lot of these people all they have is a tent and that’s one room…so it's claustrophobic and in there it's not claustrophobic and again, a lot of familiar faces.”*

These reflections illustrate how belonging is reinforced through routine, shared experiences, and the familiarity of both the people and the environment. Unlike other transient spaces, this service fosters a stable and predictable community dynamic that many participants likened to family. This combination of safety, familiarity and inclusivity fostered a strong sense of belonging amongst regular attendees.

## Discussion

Drawing on the perspectives and experiences of regular clients of the RC, the results of this study highlight the specific factors that foster regular attendance and engagement with the two supervised consumption spaces and the range of other services available on-site. Atmosphere was central to attendance; our results speak to the specific manifestations and experiences of safety, familiarity and inclusivity within the RC which supported belonging and encouraged routine attendance. Through this analytical lens, the RC emerges as a unique site which fosters social inclusion, in the wider context of a structurally unsafe environment.

Most clients attended the site regularly, if not everyday, with the service fitting into their daily routine. Whether it was to access the harm reduction services, meals, engage with staff or simply socialize with peers, the RC was described as a space which allowed for partaking in meaningful, productive daily activities. Observational research indicates that globally, individuals in good health engage in highly routinized health behaviours [38]. Despite this, a large body of research, from a range of settings, highlights how individuals who experience homelessness, or who live in precarious socioeconomic conditions struggle to identify meaningful daily activities and subsequently organize them into routines [39]. For people experiencing homelessness or vulnerable housing, a range of structural barriers impede capacity to maintain continuity with occupations and interests, including limited financial resources, social isolation, and lack of transportation [40, 41]. Others note how the majority of people’s time is occupied by surviving, and as daily challenges vary it can be difficult to incorporate wellbeing or other activities alongside [42]. Simpson et al. [39] explain that for people experiencing homelessness, daily routine encompasses *“searching for a physical space and then negotiating ways to remain in that space without being forcibly removed”* (p. 205). By acting as a space of safety to engage in productive activities, socialize, access basic necessities and build a productive routine, our findings suggest that harm reduction spaces like the RC act as a powerful anchor and countering force to the structural barriers to stability experienced by similar populations in other settings [43–45].

Location and the diversity of harm reduction services offered in the RC emerged as important in encouraging regular service attendance. Prior research has identified that proximity of SCSs to where people live, work and access drugs is a key service facilitator [46–48]. Service location and distance to the location of people's other daily activities (residence, employment etc.) has also been noted as of importance in relation to other drug services such as methadone clinics [49, 50]. Our findings highlight how proximity to other essential services such as pharmacies or shelter facilities also ensured the RC was easily accessible. Some participants also noted the RC being slightly removed from the core of the DTES as a positive. The urban drug scene of the DTES has been identified as an important risk environment that negatively shapes the health of its community members [51]. Social and cultural capital operating specifically outside the centre of the DTES, including supportive networks of friends, social services, drug treatment services and harm reduction services have similarly been identified as positive determinants of health and social well-being [52, 53]. These resources can promote positive behaviour change [24].

Participants affirmed the importance of both the diversity and quality of the harm reduction services on offer at the RC. In recent years the drug scene of the DTES has shifted from a predominance of injecting to smoking [54–56]. As a result, most fatal overdoses in this neighbourhood now occur from smoking versus any other mode of consumption [57]. The inclusion of interventions which accommodate different forms of drug consumption to reflect the evolving drug scene was a key feature that differentiated this SCS to others in the DTES. Beyond the immediate health, public health and public order impacts of broadening the scope of harm reduction provision [13], providing services for people who smoke drugs signals that the RC is a service and environment which is welcoming, non-judgmental and supportive of all people who use drugs and are at risk of overdose in the DTES.

Above all ‘atmosphere’ was frequently repeated as what set the RC apart from other local services and as the primary determinant of regular attendance. To better understand this, we specifically coded the various ways participants described this term. Participants emphasized the safety offered by the service, both in relation to the ability for the supervised consumption services safeguarding their lives, but also safety from the interpersonal (physical violence) and structural violence (criminalization and apprehension from police) experienced on the street. Beyond physical and structural safety, this term was used to describe the emotional state experienced in the RC. For example, participants valued the staff’s ability to quickly resolve conflicts, which contrasted sharply with other local services where violent incidents could escalate without effective intervention. This ability to manage conflict and maintain order was described as crucial for fostering an environment in which clients feel safe, respected, and able to access services without fear of harm or disruption. These findings support previous descriptions of safety within the SCSs; that conflict cannot necessarily be avoided, but can be managed well [15, 28, 58–60].

The role of ‘familiarity’ was also frequently described as a key contributor to the positive atmosphere of the RC. When probing these comments deeper, participants described an appreciation for the predictability of the environment, in service operations and through their relationships with staff, peers and other clients. This sense of familiarity, with peers, created a foundation of trust that facilitated engagement [21, 61–63]. Research from SCS as well as other health and social care settings suggests that trust in staff, along with continuity of care and relationships, is a significant predictor of service retention [64–67]. The familiarity with which clients engaged with staff, peers and the service was frequently recorded in the fieldnotes and was specifically noted by clients through the use of terms such as ‘family’, ‘second home’ and ‘community’ to describe the meaningful social connections formed and sustained through the service. In previous work, we have hypothesized that this sense of belonging facilitates broader social inclusion outcomes [28], though this finding is yet to be discussed in-depth in the empirical literature and merits further investigation. Additionally, participants explained that by being in a safe and familiar setting, they were able to maximize the aspects of pleasure associated with their drug use as opposed to rushing or experiencing stress during consumption as can often be experienced in street-based settings (e.g. [48, 68, 69]). The importance of accounting for the role of pleasure within the context of drug use has been noted by other scholars who emphasize the potential role in increasing service engagement [70–72].

Finally, inclusivity emerged as a core value associated with the atmosphere of the RC. Participants repeatedly emphasized the welcoming, non-judgmental nature of the service alongside several distinct markers of inclusivity. Firstly, unlike some of the faith-based organisations in the DTES, participants felt like the RC was explicitly free from religious or political agendas. All facilities within the service were open to everyone, including people with pets, couples and people who did not use drugs (eg., to access food, the common room and hygiene facilities). The mission of the RC is to support all local community members. As such, despite most clients using drugs, all described benefits resulting from the policies of inclusivity. Inclusivity has been noted by several other scholars as a key service facilitator in the nature and broader policy orientation of harm reduction services and where they are located [14, 73–76].

The combination of safety, familiarity and inclusivity fostered an environment and an experience with the service that cultivated a strong sense of belonging amongst regular service attendees with regards to the service and its community. Scher et al. [28] outline in depth the theoretical basis for the emergence of social inclusion in the context of integrated SCS services. The manifestation of this theoretical finding was evident in the narratives of RC clients. Indeed, people described a strong sense of acceptance, connection, recognition and both physical and political safety in relation to the RC and its community [77, 78]. Although a relatively unexplored outcome within the context of harm reduction services, such spaces of belonging work to counteract the isolation, alienation and stigma experienced by many structurally vulnerable and socially marginalised people who use drugs [6]. By providing a space where individuals can form meaningful social connections, access robust auxiliary health and social support, and experience a sense of community, harm reduction programs like the RC can contribute to long-term positive outcomes, including increased positive health and social outcomes and ultimately improved well-being [14, 79].

### Policy implications

This study raises several important policy considerations and implications for the design and delivery of SCSs and harm reduction services like the RC. The role of integrated SCSs in fostering daily routine and meaningful social connections and activities (e.g., peer work), highlights their broader value beyond the immediate harm reduction, biomedical, public health outcomes typically cited as justifications for their implementation. As such, at local, provincial and national levels, where possible, policymakers should prioritize integrating auxiliary services (eg., food, hygiene facilities etc.) into low-barrier SCSs; including through ensuring consistent funding and regulatory support for such services. The clear significance of the location of the RC (as being removed from the core of the DTES) as a determinant of service attendance underscores the need for thoughtful urban planning to ensure SCSs are accessible yet situated in areas that promote a sense of safety for potential clients.

As noted by other scholars [57, 68, 69, 80], our findings demonstrate the need to support people who smoke drugs as a means of ensuring harm reduction provision supports all people at risk of overdose. Despite its clear importance, ‘atmosphere’ as a theoretical concept within the context of SCSs remains relatively unexplored. Fostering a safe, familiar, inclusive SCS environment encouraged regular service attendance and ultimately increased positive outcomes for clients. In addition to increasing the number of peer staff to promote such service characteristics, additional low-barrier policies [81] such as the inclusion of people with pets, couples and people who are not at a service explicitly to use the supervised consumption services but who may benefit from the other on-site auxiliary services should be encouraged.

Many SCS evaluations to date have been quantitative, with cross-sectional and cohort study designs employed to isolate certain elements of service delivery as a means of measuring effect [17]. The recognition of the importance of atmosphere in facilitating service engagement and continuation suggests that perhaps additional interpersonal, relational core outcomes [42] should be investigated, along with the prioritization of more qualitatively driven methods of data collection.

### Limitations and future research direction

This study has several limitations. Firstly, the findings derive from a particular demographic of clients and may not be representative of the entire sample (eg., in the ‘routine and regular attendance theme’ all quotes come from white males). Additionally as a large portion of the client group were regular, routine attendees, we may not have considered the impact this could have had on clients who are new to the space. A sense of protectionism from the regular client group may warrant further investigation. Finally, although we attempted to mitigate this issue through the triangulation of qualitative data, a longer form ethnography has the potential to capture deeper insights related to the social dynamics of the facility. Based on the novel characterization of ‘atmosphere’ as a key component of regular SCS attendance, future research should look to empirically examine the core components—safety, familiarity and inclusivity–through other methodological approaches and over a longer period of time. Although theorized here in relation to a harm reduction service, in developing this concept further, it may arise as transferable to other health and social care settings.

### Conclusion

The findings of this study highlight several important factors related to the determinants of regular service attendance of integrated SCSs like the RC. Firstly, the service had a regular clientele, many who attended daily. Regular service attendance was credited to four distinct factors: (1) the accessible location, (2) the on-site auxiliary services, (3) the diversity of harm reduction provision and (4) the atmosphere. Exploring the concept of atmosphere in more depth revealed it was characterized by the safety, familiarity and inclusivity experienced. In addition to highlighting important factors which could increase access to SCSs in other settings, these findings highlight the success of integrated SCSs in offering support which addresses not just the immediate health and harm reduction needs of clients but also important social needs which foster stability. Future research should explore how these social dimensions of harm reduction services influence broader, long-term outcomes and how they can be more effectively incorporated into the design and implementation of SCS and in other contexts.

## Data Availability

Data can be made available upon reasonable request.
